# An Atomic Force Microscope with Dual Actuation Capability for Biomolecular Experiments

**DOI:** 10.1038/srep27567

**Published:** 2016-06-07

**Authors:** Semih Sevim, Naveen Shamsudhin, Sevil Ozer, Luying Feng, Arielle Fakhraee, Olgaç Ergeneman, Salvador Pané, Bradley J. Nelson, Hamdi Torun

**Affiliations:** 1 Department of Electrical and Electronics Engineering, Bogazici University, Bebek 34342 Istanbul, Turkey; 2 Multi Scale Robotics Lab, Institute of Robotics and Intelligent Systems, ETH Zurich, 8092, Zurich, Switzerland; 3 Aeon Scientific AG, Schlieren, 8952, Zurich, Switzerland; 4 Center for Life Sciences and Technologies, Bogazici University, Kandilli 34684 Istanbul, Turkey

## Abstract

We report a modular atomic force microscope (AFM) design for biomolecular experiments. The AFM head uses readily available components and incorporates deflection-based optics and a piezotube-based cantilever actuator. Jetted-polymers have been used in the mechanical assembly, which allows rapid manufacturing. In addition, a FeCo-tipped electromagnet provides high-force cantilever actuation with vertical magnetic fields up to 0.55 T. Magnetic field calibration has been performed with a micro-hall sensor, which corresponds well with results from finite element magnetostatics simulations. An integrated force resolution of 1.82 and 2.98 pN, in air and in DI water, respectively was achieved in 1 kHz bandwidth with commercially available cantilevers made of Silicon Nitride. The controller and user interface are implemented on modular hardware to ensure scalability. The AFM can be operated in different modes, such as molecular pulling or force-clamp, by actuating the cantilever with the available actuators. The electromagnetic and piezoelectric actuation capabilities have been demonstrated in unbinding experiments of the biotin-streptavidin complex.

Atomic force microscopy (AFM) is best known for high-resolution imaging, but it is also a powerful tool for sensitive force measurements. The technology has been demonstrated successfully for various exciting biomolecular measurements, such as receptor/ligand interactions and protein folding/unfolding at single-molecule level^1^,^2^,^3^,^4^. Structure, function, and energy landscape of biomolecules can be investigated via force spectroscopy experiments. The method is based on actuation of a functionalized microcantilever with attached biomolecules over a functionalized sample surface while monitoring the deflection of the cantilever, which is a measure of the force on the biomolecules. The cantilever is actuated over a wide range of speeds to obtain the energy landscape in detail. At low actuation speeds, drift in the system induces spurious deflection on the cantilever and adversely affects the measurements^5^,^6^. On the other hand, the dynamics of the actuator and the cantilever determine the attainable actuation speed. A fundamental limit for immersed cantilevers at high speeds is the hydrodynamic drag^7^,^8^. Cantilever geometry determines the effect of hydrodynamic drag. Smaller cantilevers tend to have less hydrodynamic drag as compared to larger ones^9^. In addition to hydrodynamic drag, indirect actuation of the cantilever using a large-scale piezo actuator has detrimental effects. In a conventional AFM setup, a large piezo actuates the cantilever through its holder and this excites many other modes in the complete mechanical system^10^,^11^. The resonant frequencies of the large mechanical assembly of the actuator and the holder are usually smaller than the resonant frequency of the cantilever. This sets a practical limitation for actuation at high speeds. Direct actuation of cantilevers in liquid using magnetic^12^,^13^, photothermal^14^, acoustic radiation pressure^15^ and capacitive methods^16^,^17^ have been proposed to overcome this problem. A magnetic actuator can be integrated into a conventional AFM setup by coupling an AFM head with an electromagnet or a permanent magnet. Magnetic cantilevers^18^,^19^, standard cantilevers attached with magnetic particles^13^,^20^,^21^ or current-carrying cantilevers^22^,^23^ can be used in these setups. Mechanical modification of a conventional AFM head makes it possible to add magnetic actuation capability. Rapid prototyping techniques are appealing for constructing customized AFM setups because they simplify the development of such setups according to user needs. Among different techniques, selective laser sintering (SLS) was previously used to develop a new AFM head for force spectroscopy applications^24^. The manufacturing technique was demonstrated to produce an AFM setup with similar performance levels as compared to traditional manufacturing methods.

In this paper we present an AFM setup with dual actuation capability for biomolecular experiments. The cantilever can be actuated using a conventional piezotube actuator or by a FeCo core-based electromagnet integrated to the head. The latter requires a magnetic microcantilever for high-force magnetic actuation capability. We optimized the AFM setup for force spectroscopy experiments that require an actuation range of several micrometers and an actuation bandwidth of a few kilohertz. The electromagnet design allows us to achieve a large displacement range and high frequency operation simultaneously. Previously, magnetic actuators for standard cantilevers attached with magnetic particles have mainly been employed for tapping mode imaging where the displacement amplitude of the cantilevers is limited to tens of nanometers^13^,^20^,^21^. The limited range of displacement limits the application of magnetic actuation for force spectroscopy experiments. Nevertheless, the method has been employed to provide force modulation with small oscillation amplitudes for force spectroscopy experiments^25^. In addition, the actuation bandwidth of the actuator is limited significantly when a coil of an electromagnet is used together with a core that is needed to increase the displacement range^26^,^27^. So, coils without cores are usually used to increase bandwidth at the expense of displacement range^20^,^21^. Excessive heating imposes another limitation for the utilization of magnetic actuators, which can provide large displacement range at high bandwidth. This may become especially important for current-carrying cantilevers that experience Joule heating^22^,^23^. We have developed a closed-loop temperature stabilizer that controls the temperature of the samples within 0.1 °C. The coil and the core are cooled using a dual cooling stage comprising a water-based cooler in addition to a thermoelectric cooler for fine-regulation of temperature.

The prototype was manufactured using jetted-photopolymers. This method of rapid prototyping allows three-dimensional printing with a wide variety of composite materials. The AFM setup is modular and can be modified with ease. We implemented a software-based controller capable of controlling both actuators for force spectroscopy experiments.

## Results

### Characterization

**Figure 1:**
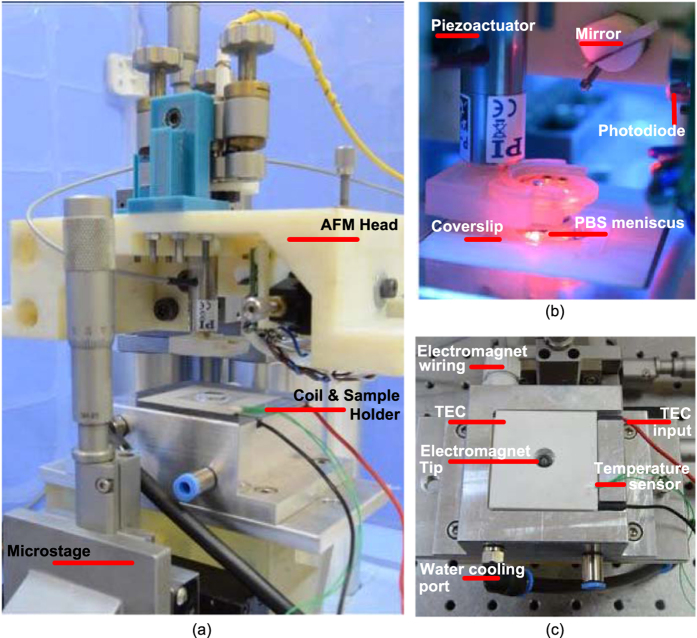
(**a**) Integrated AFM head with coil-sample holder. (**b**) The manufactured AFM head with commercial elements, (**c**) Coil-Sample holder.

Our AFM was designed with a focus on increased hardware and software modularity for biomolecular applications. The modular design consists of the AFM head with piezoactuation and the electromagnetic system as shown in Fig. 1. The details of the design and the realization of the setup are explained in detail in the methods section.

**Figure 2:**
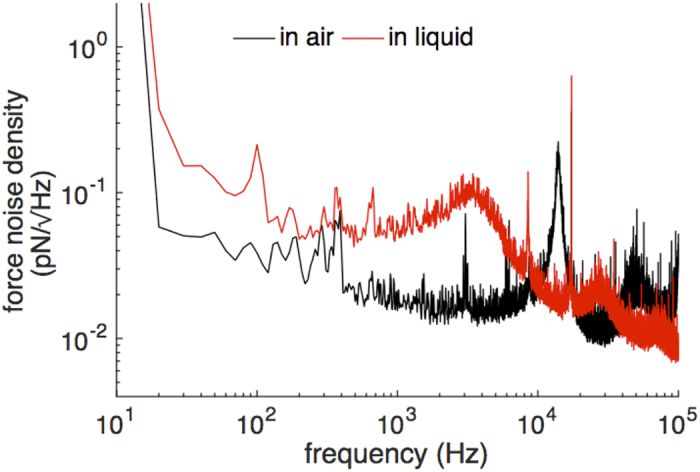
Power spectral density of the deflection signal acquired using a commercial AFM cantilever in air and in liquid.

We have carried out a noise analysis in detail to evaluate the performance of the prototyped AFM. Figure 2 presents a typical frequency spectrum of the vertical deflection signal obtained in our setup in air (black line) and in DI water (red line) for a commonly used cantilever (SNL10-D cantilever, Bruker, USA). We calibrated the cantilever for its spring constant and obtained the deflection sensitivity before we acquired the noise spectra. The signals were acquired with a bandwidth of 100 kHz. The cut-off frequency of the photodiode we use for the deflection signal is 250 kHz. The optical power of the laser for this measurement was 5 mW. The broad peaks at about 18 kHz in air, and at about 4 kHz in water are the resonant frequencies of the basic vibration modes of the cantilever due to the Brownian motion. The resonant frequencies for the second mode of the cantilever are also seen in the acquired noise spectra. Integrated noise levels of 1.82 pN in air and 2.98 pN in liquid with a detection bandwidth of 1 kHz were experimentally demonstrated with the cantilever. The noise levels are limited with the thermal noise of the cantilever.

**Figure 3:**
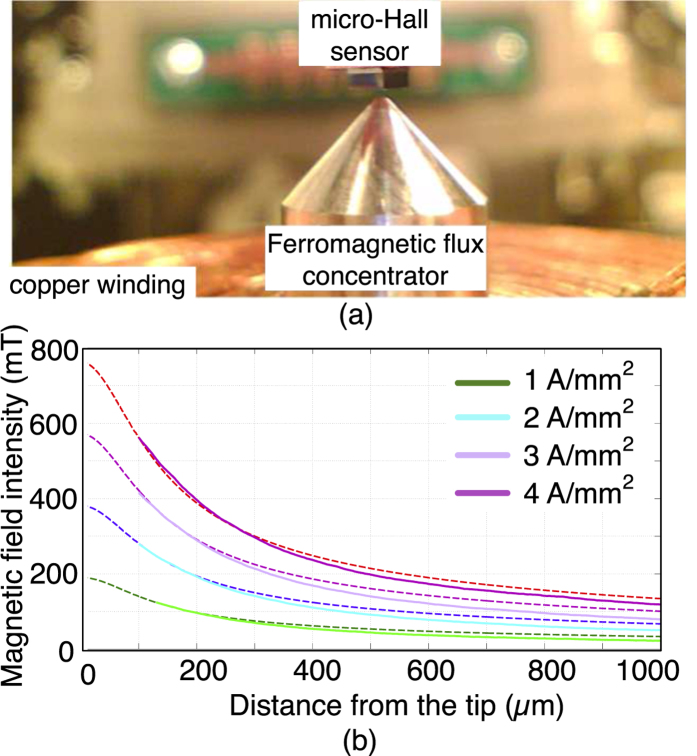
(**a**) The electromagnet is shown along with the micro-hall sensor which was used for characterizing the magnetic flux density. (**b**) The experimentally measured vertical magnetic flux density B_z_ is compared with FEM simulations at various current densities.

The DC magnetic field generation capability of the electromagnet was quantitatively characterized with a micro-hall sensor (Nanomagnetics Ltd, Ankara, Turkey) having an active sensing area of 1 × 1 μm^2^ (see Fig. 3a). The magnetic flux density, Bz was measured at various input current densities. The axial decay of the flux density from the tip of the electromagnet was found to be an excellent match with the FEM design simulations (Fig. 3b). We achieved a flux density of over 0.55 Tesla at a working distance of 100 μm from the pole-piece.

**Figure 4:**
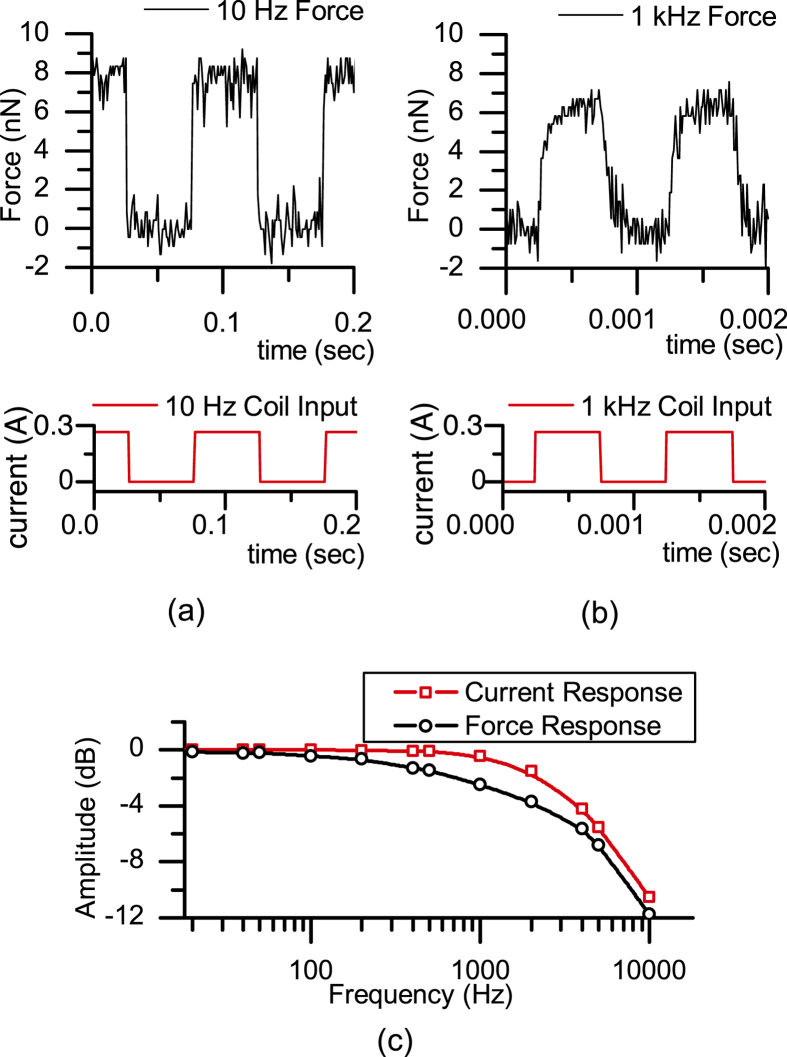
Magnetic actuation of a commercial MFM cantilever (MESP, Bruker Probes) in air (**a**) at 10 Hz (**b**) at 1 kHz using rectangular voltage drive signals. (**c**) Force and current response graph with respect to frequency. For (**a–c**), the actuating electromagnet employed a FeCo core-element.

We have characterized the AC actuation forces of the electromagnet using a commercial MFM cantilever (MESP, rectangular, Bruker Probes) placed on the cantilever holder with the electromagnet placed under the AFM head as shown in Fig. 1a–c. The Si cantilever has a hard magnetic CoCr coating and its stiffness was calibrated as 1.12 N/m. We drove the amplifier of the electromagnet with an input voltage signal to keep the output current of the amplifier constant. The electromagnet was driven with a square wave up to 10 kHz. In Fig. 4a,b, 10 Hz and 1 kHz magnetic actuation of a commercial MFM cantilever in air are shown, respectively. At 1 kHz, a square wave with 300 mA amplitude results in a magnetic force of 7.5 nN on the MFM cantilever. Also, the frequency of the system was obtained by applying sinusoidal input voltage signal to the electromagnet by sweeping the frequency. The response for the measured current on the coil and the force measured on the cantilever is plotted in Fig. 4c. The force response decays faster than the current response because of the lowering of effective permeability due to hysteretic and eddy current losses in the core material. The bandwidth of the force response can be significantly increased using the ferrite core, which has lower hysteretic and eddy current losses, albeit at the cost of overall reduced force, because of its lower saturation induction (Ms ~ 0.4 T).

**Figure 5:**
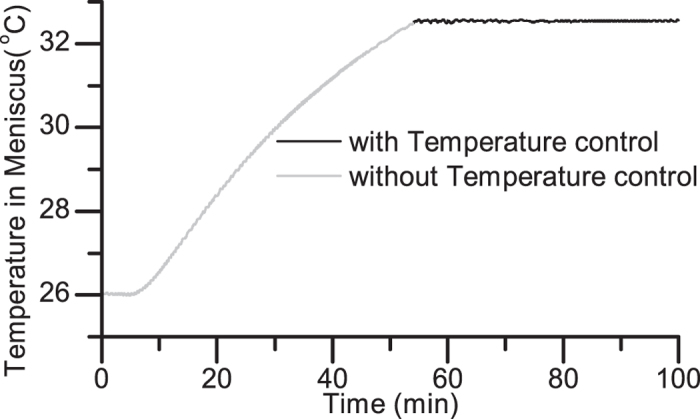
Temperature trace of an immersed cantilever actuated using the electromagnet. The temperature starts rising when the electromagnet is turned on at t = 5 min. The temperature controller is turned on at t = 53 min to stabilize the temperature of the sample.

Biomolecular interactions are extremely sensitive to environmental temperature and fluctuations. Thermal drift induces cantilever deflection and changes the zero-force level, which are detrimental for force spectroscopy. Joule heating from the current actuation of the electromagnet can perturb the experiments. We developed a temperature control system, which can regulate the temperature fluctuations from the electromagnet operation in our fluid environment to within 0.1 degrees. The control was achieved using sub-mm sized thermistors placed in the liquid meniscus along with a thermoelectric cooler and PID controller. Figure 5 shows how the temperature of a cantilever rises when the electromagnet is turned on with the initial temperature of the cantilever was 25.9 °C. The electromagnet was driven with a square wave with a frequency of 0.2 Hz and peak current of 1.8 A for 100 min. The temperature increased to a temperature of 32.5 °C when the temperature controller was turned on at t = 53 min.

### Biomolecular experiment

We performed biomolecular force spectroscopy experiments with the new system to probe biotin/streptavidin interactions. We used piezo actuation and magnetic actuation during the experiments. The mechanics and the dynamics of the biotin-streptavidin complex have been characterized in detail (cite: Merkel 1999; Yuan 2000). Streptavidin is a tetrameric protein that has affinity for biotin molecules. Force spectroscopy enables us to understand the formation and dissociation of biological weak bonds between single molecule pairs. We used biotin coated cantilever tips (CT.BIO, Novascan, Ames, IA USA) for the experiments. The nominal spring constant of the cantilevers is 0.01 N/m and the cantilevers were calibrated for their spring constants prior to the experiments using thermal noise method. Biotin is covalently bonded to the tip of the commercial cantilever. Biotin coated cantilevers were then brought into contact with a substrate covered with immobilized streptavidin (VXP0010, Xenoprobe). Streptavidin is uniformly coated over the surface of the substrate and the molecules are covalently bonded. The experiments were performed in 100 μL- phosphate buffered saline (PBS) at room temperature.

**Figure 6:**
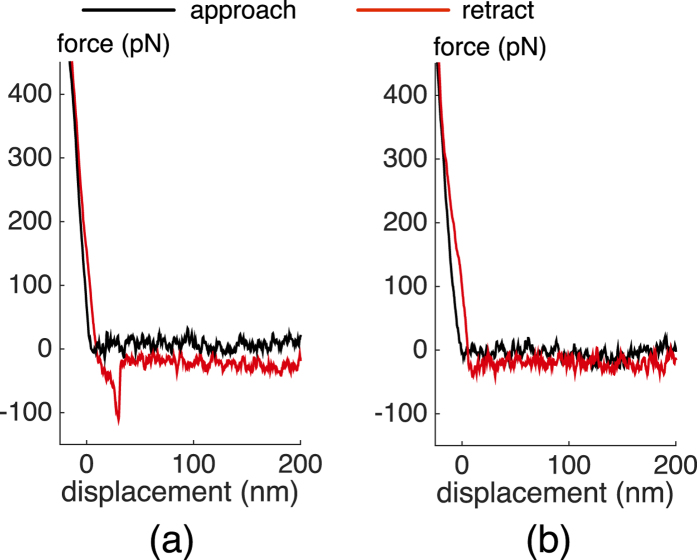
A sample force curves showing (**a**) a specific biotin-streptavidin interaction with an unbinding force of ~100 pN and (**b**) no adhesion/rupture event.

Representative AFM force curves obtained with piezo actuation are shown in Fig. 6. During the approach phase of the recording cycle (black line), the AFM tip is lowered onto the streptavidin sample. No interaction was detected between the tip and the surface until they were brought into contact. After the cantilever was pushed against the substrate to allow bond formation, the cantilever was retracted back (red line). Figure 6a shows a force curve where an unbinding force of ~100 pN was measured. The nonlinear portion of the curve towards negative forces indicates the loading of a bond between the molecules. The sudden jump indicates the rupture of a single bond and the magnitude of the vertical transition is a measure of the strength of the bonds. Figure 6b shows another force curve where no adhesion/rupture event was detected. The speed of the piezo during the measurements was 3 μm/s and the small offset between the zero-force levels between the approach and retract cycles are due to the hydrodynamic drag experienced by the cantilever. The loading rate was varied between 10^2^ and 10^6^ pN/s during the experiment, and hundreds of approach-withdrawal cycles were recorded for each loading rate.

Biomolecular pulling experiments using biotin/streptavidin pair were also performed using a commercial AFM system (Dimension Edge, Bruker Nano, Santa Barbara, CA USA) for comparison. The same type of cantilevers was used for the experiment. The cantilever was actuated against a cover slip coated with streptavidin molecules. 20 μl of streptavidin solution (10 mg/100 μl) was diluted with 50 μl of buffer solution for three times for the incubation process. The cover slips were incubated for 13 minutes at room temperature. The piezotube of the commercial AFM system was actuated at different speeds for the experiment. At each speed, approximately 100 force curves were recorded to obtain statistically significant data.

**Figure 7:**
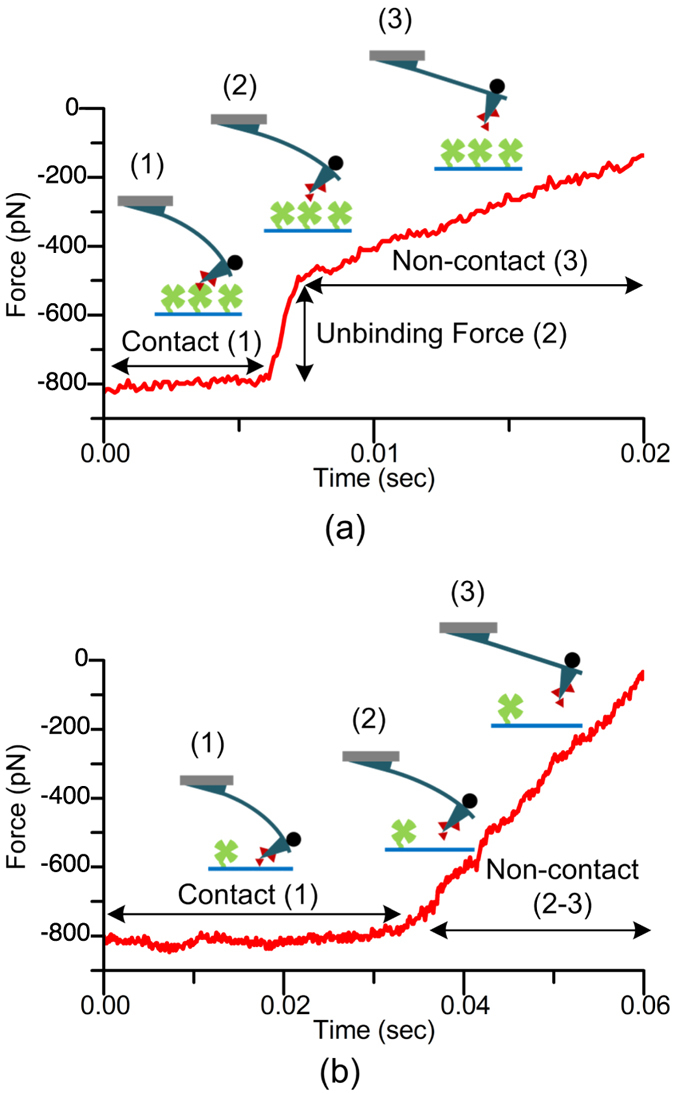
Sample force traces obtained using a magnetic bead attached cantilever actuated using the electromagnet showing (**a**) a specific biotin-streptavidin interaction with an unbinding force of 285 pN and (**b**) no adhesion/rupture event.

A ferromagnetic bead with a diameter of 30 μm was attached to a cantilever (MLCT-C, Bruker Probes) to probe the interactions between streptavidin and biotin via magnetic actuation. The cantilever was functionalized with biotin and tested against the substrate covered with immobilized streptavidin (VXP0010, Xenoprobe) in PBS. During magnetic actuation experiments, the cantilever is brought within the vicinity of the substrate and actuated using a triangular current wave applied to the electromagnet. First, the electromagnet attracts the cantilever and the cantilever comes into contact with the substrate to allow the formation of molecular bonds. The displacement of the cantilever does not change when it is in contact with the substrate. Then the electromagnet repels the cantilever away from the substrate. Figure 7a shows a typical force trace where an unbinding force of 285 pN was measured before the cantilever lost contact with the substrate. Figure 7b shows another force trace where no adhesion/rupture event was observed. The fundamental difference between the piezo and magnetic actuation methods is the way the cantilever is set in motion. The base of the cantilever is in motion during piezo actuation and it follows the actuator. The cantilever is not bent unless it is in contact with a sample surface, ignoring small bending that can be induced due to hydrodynamic drag force. On the contrary, the cantilever is pulled at its tip while its base is stationary during magnetic actuation. So, the cantilever is bent due to the force applied at its tip when it is free from a sample surface. The magnetic force cannot induce bending when the cantilever is in contact with a sample surface since its tip defines an anchor point. Nevertheless, sudden jumps indicate unbinding forces during biomolecular experiments. This behavior can be observed in Fig. 7.

**Figure 8:**
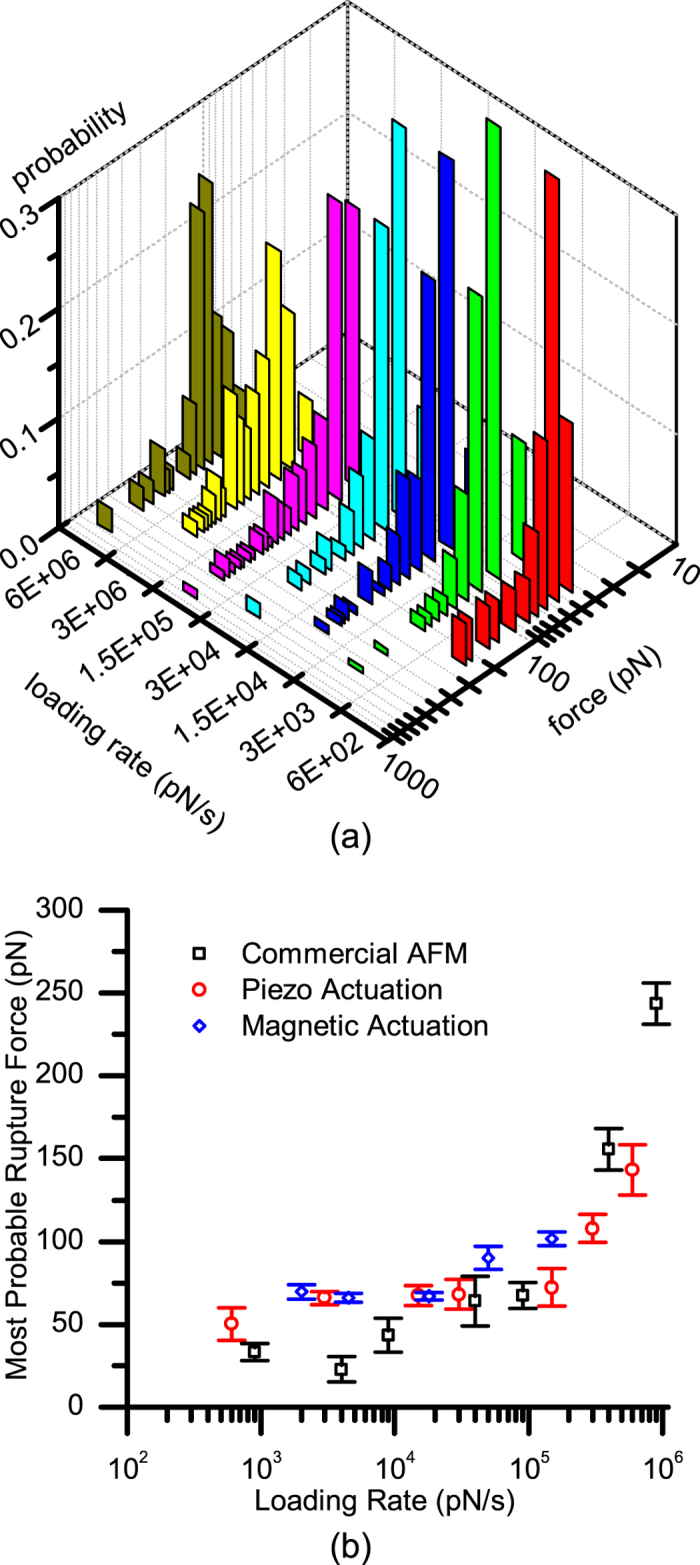
(**a**) Force and probability of interaction histograms for biotin-streptavidin interaction with respect to loading rates (**b**) Loading Rate vs. most probable rupture force obtained using piezo and magnetic cantilever actuation compared with the results of experiments obtained using a commercial AFM system.

Figure 8a shows the force histograms for the data collected. Most probable rupture force as a function of loading rate is presented along with the probability of specific events. The results of the experiments using the customized AFM setup with piezo and magnetic actuation together with the data collected using the commercial AFM setup are compared, as shown in Fig. 8b. The most probable rupture force levels corresponding to each loading rate for both experiments were calculated by fitting a Gaussian distribution curve to each data set. The results are in good agreement, validating the functionality of the new AFM setup.

## Discussion

We adopted a modular approach to the design and development of the AFM for biomolecular force spectroscopy applications. The use of jetted-polymers enables rapid design and manufacture cycles and the polymers have proven to be mechanically and thermally stable over time. The PXI embedded platform provides software and controller scalability. The flexible design approach allows system modification for customized applications. The experimentally characterized force resolution of the system is 1.82 pN in air with a 1 kHz bandwidth. This is 2.98 pN in DI water, limited with the thermal noise of commercially available cantilevers. An electromagnet was implemented to enable direct cantilever actuation. The electromagnet is placed below the sample stage within a distance of 100 μm from the cantilever. The electromagnet design is optimized for providing a large displacement range at high frequency. It is possible to apply a field of 0.55 T on the cantilever using the electromagnetic actuation capability of the system. This allows generation of force level of several nanonewtons with a bandwidth of 1 kHz that is adequate for force spectroscopy experiments. Unbinding experiments were performed on the biotin-streptavidin pair using the piezoelectric and electromagnetic cantilever actuation. Most probable rupture force as a function of loading rate were obtained using both the electromagnetic actuator and the conventional piezo actuator. The obtained data allow the determination of the parameters of the Bell-Evans model^28^. The results of molecular pulling experiments performed by the introduced system were in agreement with those obtained using a commercial AFM. We observed the values of the most probable rupture forces increase rapidly for the loading rates larger than 10^5^ pN/s. Two distinct loading rate regimes is characteristic for biotin-streptavidin pairs as this indicates the presence of at least two energy barriers^28^.

## Methods

The AFM head and the sample/electromagnet holder are portable and were manufactured using low-cost jetting of a rigid polymer (Rigid Opaque from Stratasys Ltd., MN, USA). The electromagnet enables high-force DC and AC actuation of soft and hard magnetic cantilevers. The flexible software-based controller and the user interface was implemented in National Instrument’s LabView, which runs on a Windows-based operation system (OS), as well as on a real-time OS for high-speed applications. The software module can operate the AFM in both force spectroscopy mode and force-clamp mode by monitoring the laser photodiode signal and drive the cantilever appropriately. It also provides the actuation signal for both the piezoactuator and the electromagnet. The AFM hardware assembly is placed on a vibration isolation table and an acoustic enclosure for minimal noise operation, as shown in Fig. 1a. The dual actuation capabilities of the AFM allow for a wide range of biomolecular experiments.

The 3D-printed AFM head shown in Fig. 1b comprises a fiber-pigtailed laser diode (λ = 650 nm, Oz Optics Ltd. Ottowa, Canada), which focuses the light beam onto the cantilever mounted on a piezo actuator (P-841.1, Physik Instrumente GmbH). The reflected light is directed by a mirror into a quad-photodiode array (Pacific Silicon Sensor, CA, USA). The cantilever is mounted to a commercially available liquid cell (DECAFMCH-PFT, Bruker, USA), which is attached to the piezoactuator via a 3D-printed custom adaptor. The piezoactuator has a vertical range of 15 μm. A kinematic optical mount (Newport Corporation, CA, USA) embeds the laser fiber and is used to orient and focus the laser beam onto the cantilever. The mirror directing the laser beam has a rotational degree of freedom and the photodetector is mounted on a miniature two-axis translator (Thorlabs Inc. NJ, USA). The AFM head has been designed to allow easy integration onto an electromagnetic coil system for direct cantilever actuation, in addition to other magnetic applications.

The electromagnet consists of an optimized ferromagnetic core geometry and copper winding. The design objective was to maximize the force on a magnetic film-coated or magnetic particle-attached cantilever at a working distance of 100–150 μm from the pole-piece of the electromagnet. We parameterized the geometry of the electromagnet into the radius and length of the cylindrical core, the length and the inner and outer radii of the winding, and lastly the shape of the core-tip. The parameterization allowed us to optimize the design in a finite-element based magnetostatics solver (Ansys Maxwell). The exchangeable core-piece is made of FeCo alloy (Vacoflux50^TM^) because of its high magnetization saturation (M_s_ = 2.35 T), which corresponds to higher actuation force. For high frequency operation in the tens of kilohertz range, a ferrite-core (3C85 - Ferroxcube) can be used. Since biomolecular experiments have strict requirements on temperature control and stability, we implemented a dual cooling stage comprising a thermoelectric cooler (UEPT-440-127-079E120 – Uwe Electronic GmbH) for fine-regulation of temperature on top of a water-based cooling system (Fig. 1c).

The AFM system is controlled using a PXI embedded controller (NI PXI-8102, National Instruments, TX, USA) via an expansion card with 8 analog inputs (F_s_ = 1.25 MS/s), 2 analog outputs (F_s_ = 3.33 MS/s) and 24 digital inputs/outputs. The controller and the user interface are programmed in NI-LabView environment. The main function of the controller is to drive the piezotube with customizable voltage waveforms, record the cantilever deflection, and record data.

The controller is capable of performing molecular pulling and force-clamp experiments. Force-clamp mode controls the cantilever in a feedback-loop to keep the force on biomolecules the same. The PID controller can be programmed to drive both the piezo and the coil amplifier of the AFM system. A loop speed of 50 kHz has been achieved using the NI Real-time operation system (RTOS) with the controller, while the loop speed drops to 7 kHz on a Windows-OS based system. Controller speed for that system is limited by the CPU speed and the buffer size of the computer and the DAQ card. The P, I, and D parameters are tuned in real-time by observing the deflection signal of the cantilever to optimize the controller.

## Additional Information

**How to cite this article**: Sevim, S. *et al.* An Atomic Force Microscope with Dual Actuation Capability for Biomolecular Experiments. *Sci. Rep.* **6**, 27567; doi: 10.1038/srep27567 (2016).
